# Low Neutralizing Antibody Titers against the Mu Variant of SARS-CoV-2 in 31 BNT162b2 Vaccinated Individuals in Colombia

**DOI:** 10.3390/vaccines10020180

**Published:** 2022-01-24

**Authors:** Diego A. Álvarez-Díaz, Ana Luisa Muñoz, Pilar Tavera-Rodríguez, María T. Herrera-Sepúlveda, Hector Alejandro Ruiz-Moreno, Katherine Laiton-Donato, Carlos Franco-Muñoz, Dioselina Pelaez-Carvajal, Diego Cuellar, Alejandra M. Muñoz-Suarez, Marisol Galindo, Edgar J. Arias-Ramírez, Marcela Mercado-Reyes

**Affiliations:** 1 Grupo Genómica de Microorganismos Emergentes, Dirección de Investigación en Salud Pública, Instituto Nacional de Salud, Av. Calle 26 No 51-20, Bogotá 111321, Colombia; mherrera@ins.gov.co (M.T.H.-S.); ha.ruiz75@gmail.com (H.A.R.-M.); kdlaitond@unal.edu.co (K.L.-D.); cfranco@ins.gov.co (C.F.-M.); dpelaez@ins.gov.co (D.P.-C.); mgalindo@ins.gov.co (M.G.); mmercado@ins.gov.co (M.M.-R.); 2 Fundación Banco Nacional de Sangre Hemolife, Bogotá 110911, Colombia; ana.munoz@hemolifeamerica.org; 3 Dirección de Investigación en Salud Pública, Instituto Nacional de Salud, Bogotá 111321, Colombia; ptavera@ins.gov.co (P.T.-R.); dicuell@gmail.com (D.C.); 4 Grupo de Parasitología, Dirección de Investigación en Salud Pública, Instituto Nacional de Salud, Bogotá 111321, Colombia; 5 Dirección de Producción, Instituto Nacional de Salud, Bogotá 111321, Colombia; ammunoz@ins.gov.co (A.M.M.-S.); earias@ins.gov.co (E.J.A.-R.)

**Keywords:** COVID-19, spike protein, SARS-CoV-2 variants, neutralizing antibodies, Mu (B.1.621) variant, gamma (P.1) variant

## Abstract

Global surveillance programs for severe acute respiratory syndrome coronavirus 2 (SARS-CoV-2) are showing the emergence of variants with mutations in the spike protein. Genomic and laboratory surveillance are important to determine if these variants may be more infectious or less susceptible to antiviral treatments and vaccine-induced antibodies. Three of the most predominant SARS-CoV-2 variants in Colombia during the epidemiological peaks of 2021 were isolated: Mu, a variant of interest; Gamma, a variant of concern; B.1.111, which lacks genetic markers associated with greater virulence. Microneutralization assays were performed by incubating 120 mean tissue culture infectious doses (TCID50) of each SARS-CoV-2 isolate with five two-fold serial dilutions of sera from 31 BNT162b2-vaccinated volunteers. The mean neutralization titer (MN50) was calculated by the Reed–Muench method. At the end of August, Mu represented 49% of coronavirus disease 2019 (COVID-19) cases in Colombia, followed by 25% of Gamma. In contrast, B.1.111 became almost undetectable. The evaluation of neutralizing antibodies suggests that patients vaccinated with BNT162b2 generate neutralizing antibody titers against the Mu variant at significantly lower concentrations relative to B.1.111 and Gamma. This study shows the importance of continuing surveillance programs of emerging variants, as well as the need to evaluate the neutralizing antibody response induced by other vaccines.

## 1. Introduction

Between October and December 2020, genomic epidemiology data from the emerging SARS-CoV-2 lineages B.1.1.7 (Alpha), B.1.351 (Beta), and P.1 (Gamma) suggested a significant association with increased transmissibility, and, consequently, a global risk to public health [1]. With this evidence, the Centers for Disease Control and Prevention (CDC) and the World Health Organization (WHO) established a hierarchical classification system to distinguish the emerging variants of SARS-CoV-2 into a variant of interest (VOI) or variant of concern (VOC) [2]. Mutations in the spike RBD are present in the different SARS-CoV-2 variants, and have been mainly associated with resistance to neutralizing activity [3,4]. To date, the WHO has designated five VOCs (Alpha, Beta, Gamma, Delta, and Omicron) and five VOIs (Eta, Iota, Kappa, Lambda, and Mu). However, a VOI might escalate to a VOC if, besides the presence of genetic markers associated with higher virulence, supported by epidemiological data suggesting that it is an emerging risk to global public health, there is solid evidence of negative impacts on public health, including increased transmissibility, virulence, and decreased effectiveness of therapeutic measures, vaccines, or diagnostics [5].

The new SARS-CoV-2 B.1.621 lineage, detected in January 2021, was proposed as a variant of interest (VOI) following the SARS-CoV-2 genomic surveillance in Colombia between December 2020 and April 2021 [6]. By the end of August 2021, the WHO confirmed the VOI status of this lineage and assigned it the “Mu” letter of the Greek alphabet [5]. Since its detection, the Mu variant has spread to 43 countries, with a higher presence in the British Virgin Islands, Colombia, Dominican Republic, Ecuador, and Haiti [7]. In Colombia, B.1.621 spread from the Caribbean region to the rest of the country within six months, and by the end of August, it was the predominant lineage [8]. The P.1 (Gamma) lineage was identified in November 2020 in Brazil and designated as a VOC in January 2021 [5]. This lineage spread worldwide and diverged into 22 sub-lineages, with a higher prevalence in American countries, including Haiti, Brazil, Venezuela, and Trinidad and Tobago [9]. In Colombia, this lineage entered from the Amazon border with Brazil at the beginning of January 2020 and dispersed throughout the country, Gamma displaced all the dominant lineages except for Mu, during the third epidemic peak [4]. The B.1.111 lineage was identified in the United States (US) by March 2020, then spread worldwide, with a higher prevalence in Venezuela, Guyana, Trinidad and Tobago, and Colombia, until its extinction at the end of August 2021 [10]. In Colombia, this lineage had a prevalence of around 40% during the first and second epidemic peaks [11], where it diverged into a few sub-lineages, including one carrying mutations on the spike (S) protein, with evidence of escape from neutralizing antibodies [4,6].

Several vaccine platforms have been granted worldwide, including the Pfizer-BNT162b2, which is an mRNA-based vaccine that encodes the SARS-CoV-2 full-length S gene [12]. Early multinational clinical trials suggested efficacy of 95% in the prevention of COVID-19 before the emergence of SARS-CoV-2 VOCs and VOIs [13]. However, in Pfizer-BNT162b2-vaccinated individuals, there is evidence of a significant reduction in the neutralization antibody titer against the Alpha, Beta, and Delta VOCs by 2.6, 4.9, and 5.8-fold, relative to the wild-type, respectively [14].

Preliminary studies assessing the sensitivity of the Mu variant to antibodies induced by the BNT162b2 vaccination using pseudoviruses and replication-competent SARS-CoV-2 yielded contradictory results [15,16]. In this study, we determined the neutralizing antibody titers in BNT162b2-vaccinated individuals against SARS-CoV-2 isolates from the Mu, Gamma, and B.1.111 lineage, using microneutralization assays.

## 2. Materials and Methods

This study was performed according to the ethical principles of the Declaration of Helsinki. It was approved by the Ethics Committee of the Colombian National Health Institute (CEMIN)-10-2020. All participants responded voluntarily to a written informed consent formulary.

The spatiotemporal distribution of SARS-CoV-2 lineages circulating in Colombia between January and August 2021 was determined following the National Program for the Genomic Characterization of SARS-CoV-2 based on representativeness and virologic criteria for probabilistic sampling [6,17]. Then, we isolated the three most predominant SARS-CoV-2 lineages during this period to evaluate the mean titer of neutralizing antibodies by microneutralization assays, as previously described [4]. Briefly, two-fold serial dilutions ranging from 1:4 to 1:2460 of serum samples were incubated with 120 median tissue culture infectious doses (TCID50) of each variant. We tested the sera from volunteers, which was collected between 18 and 22 weeks after receiving the second dose of the BNT162b2 coronavirus disease 2019 (COVID-19) vaccine. A panel of human sera from 31 volunteers (3 males and 28 females) vaccinated with BNT162b2 (age range, 23–62 years) was evaluated.

Individuals with a previous or current SARS-CoV-2 infection or an infection during clinical follow-up, or those with the presence of total antibodies against SARS-CoV-2 at the time of the first dose of vaccine administration, were excluded. The protective neutralizing antibody titer that prevented cytopathic effect in 50% of the wells (MN50) was calculated by the method of Reed and Muench [18]. All neutralization assays with infectious SARS-CoV-2 viruses were conducted in a biocontainment laboratory.

Sera were first screened for the absence of IgG anti-nucleoprotein antibodies using a qualitative ELISA (ID Screen SARS-CoV-2-N IgG Indirect, ID Vet). Subsequently, to determine the concentration of anti-spike IgG antibodies, samples were tested with the SARS-CoV-2 IgG assay (sCOVG) on the ADVIA Centaur XPT platform (Siemens) using the kit cut-off value (reactive ≥ 1.0 U/mL); the results were expressed in binding antibody units per milliliter (BAU)/mL using the conversion factor of 21.8, as determined by the manufacturer and based on the WHO first standard 20/136 [19].

## 3. Results

### 3.1. Mu and Gamma Variants Dominated the Third Epidemic Peak of SARS-CoV-2 in Colombia

**Figure 1:**
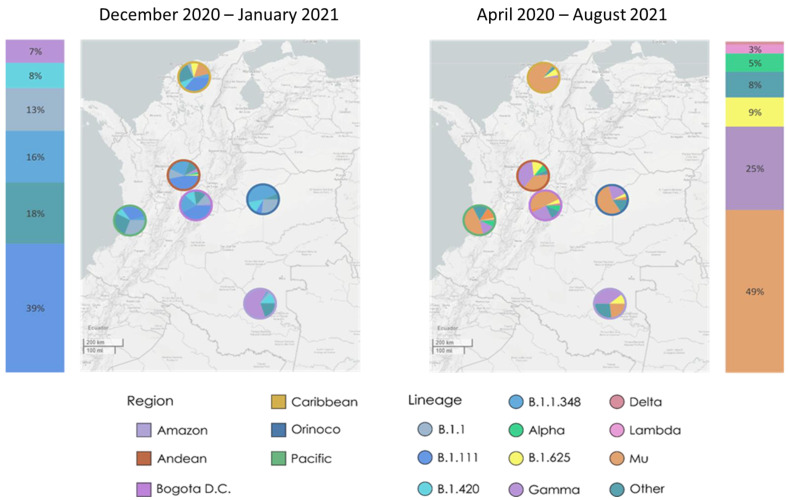
Spatiotemporal distribution of the most representative SARS-CoV-2 lineages in Colombia (bars) and its regions (map). SARS-CoV-2 lineage distribution in the five Colombian regions and Bogotá D.C. December 2020–January 2021 (**left**), and April–August 2021 (**right**). Ring colors represent the region. The interactive map is available at https://microreact.org/project/6GjGXeoUW7uVauMTFCFEkE/d9357c6c (accessed on 8 January 2022) [20]. Figure design was based on data by OpenStreetMap (https://openstreetmap.org accessed on 8 January 2022), CC BY-SA 2.0; ODbL. Abbreviations, D.C.: Distrito Capital (Capital District).

Three SARS-CoV-2 variants were isolated, Mu (B.1.621 lineage, GISAID ID EPI_ISL_1821065), Gamma (P.1 lineage, GISAID ID EPI_ISL_2500971), which was classified as a variant of concern (VOC) by the WHO, and B.1.111 (GISAID ID EPI_ISL_526971), which lacks genetic markers associated with greater virulence. Their profile of mutations in S and other regions is shown in Table 1. Molecular epidemiologic data allowed us to determine the spatiotemporal distribution of the most representative SARS-CoV-2 lineages in Colombia during the two epidemiological peaks of SARS-CoV-2 in 2021. The sequences obtained by probabilistic sampling indicated a significant increase in Mu (B.1.621) and Gamma (P.1), which were detected during the second peak in opposite regions of the country by the end of December 2020. While Mu was identified in the Caribbean region in the north, Gamma was identified in the Amazon region in the south. Then, by the end of August 2021, those lineages were dominant and widely distributed across the country, becoming the majority (74%) of COVID-19 cases in Colombia. Remarkably, Mu represented almost twice the sequences of Gamma and half of the total sequences of SARS-CoV-2 (49% of cases) in the country by the end of the third peak. In contrast, B.1.111 was distributed at the country level and dominated the first epidemic peak, but then became almost undetectable by the end of the third peak (Figure 1).

### 3.2. Mu and Gamma SARS-CoV-2 Variants Escape from Neutralization by BNT162b2 Vaccine Serum Samples

Anti-spike IgG antibody titers were identified in all the samples, but no anti-nucleoprotein IgG antibodies were identified, suggesting the exclusive presence of vaccination-induced antibodies.

**Figure 2:**
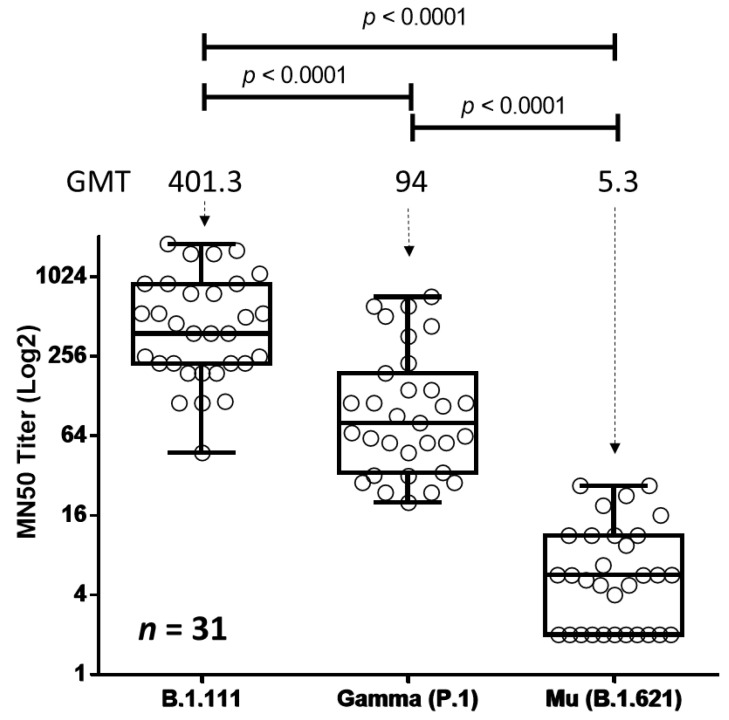
Neutralizing titers of BNT162b2-vaccinated volunteers against Mu, Gamma, and B.1.111 SARS-CoV-2 isolates. TCID50-based assays were performed by incubating 120 TCDI50 of each SARS-CoV-2 isolate with five two-fold serial dilutions of sera from BNT162b2-vaccinated volunteers. The MN50 titer was calculated by the Reed–Muench method. Statistical differences between the median values of MN50 titers against Mu, Gamma, and B.1.111 variants were determined using the Kruskal–Wallis test, followed by Dunn’s post hoc test for multiple comparisons. An arbitrary MN50 titer value of 2 was assigned to the 11 out of 31 serum samples that do not neutralize the virus at the lowest dilution (1:4). Abbreviations. *n*: number of samples, GMT: geometric mean titer, MN50: mean neutralizing antibody titer.

The serum samples exhibited robust neutralization against the B.1.111 lineage, with a geometric mean titer (GMT) of 401.3 TCID50 (Figure 2). For the Gamma variant, the GMT was 94 TCID50, which was 4.3-fold lower than B.1.111.

In contrast, for the Mu variant, the GMT was 5.3 TCID50, which was 75- and 17.7-fold lower than the B.1.111 and Gamma lineages, respectively (*p* < 0.0001). It is important to note that 11 out of 31 serum samples (35.5%) did not neutralize the virus at the dilution 1:4, which was the lowest evaluated (Figure 2).

Finally, a strong correlation was observed between the neutralizing antibody titers (MN50) and IgG anti-S antibodies (BAU/mL) for B.1.111 compared with Mu and Gamma (Table 2).

## 4. Discussion

Data from the routine genomic surveillance of SARS-CoV-2 in Colombia show the epidemiologic dynamics of SARS-CoV-2 lineages in the country during the second and third epidemiological peaks between January and August 2021 [6,8]. This suggests significant community transmission of the emerging variants Mu (B.1.621) and Gamma (P.1), which led to the rapid dispersal from the Caribbean and Amazon regions and displacement of the dominant lineages by August 2021. Furthermore, according to the INS epidemiological surveillance system, the number of confirmed cases of SARS-CoV-2 during the second epidemiological peak was almost double that reported during the first peak [21].

Hence, as the Mu and Gamma variants represented 49% and 25%, respectively, of the SARS-CoV-2 sequences analyzed in this study [8], it is probable that these variants are associated with the increase in the number of cases during the third epidemiological peak in Colombia, although the greater representativeness of Mu potentially implies the greater epidemiological impact of this variant.

Each day there is more evidence about vaccine-elicited antibodies showing a reduction in the neutralizing titer against variants such as Alpha, Beta, Gamma, and Delta [14,22]. In line with this, studies on neutralizing antibodies in SARS-CoV-2 convalescent and BNT162b2-vaccinated individuals against Omicron (B.1.1.529), a VOC identified in South Africa at the end of November 2021, reported a reduction in the MN50 titer by 43- and 127-fold, relative to the Wuhan-hu-1 strain [23]. Mutations in the spike RBD have been defined as one of the causes of this resistance [22,23]; however, it is not clear how the subsequent amino acid changes can be involved in this response [24].

In this study, a 4.3-fold decrease in the neutralization of Gamma relative to the B.1.111 variant was observed. Similar results were found in previous studies reporting a 3- to 5.12-fold reduced sensitivity of the variant Gamma to serum from individuals vaccinated with the Pfizer vaccine BNT162b2, using pseudovirus bearing the Gamma or wild-type SARS-CoV-2 S protein, with D614G exchange [15,25].

On the other hand, the sera from BNT162b2-vaccinated volunteers exhibited a robust decrease in the neutralization of Mu by 75.7- and 17.7-fold, relative to B.1.111 and Gamma, respectively. While this was consistent with the report by Uriu et al., who reported lower neutralization titers against Mu relative to a parental D614G variant and Gamma [23], the magnitude of the difference was more noticeable in the present study. By contrast, Messali et al. reported a slightly lower neutralization titer in the sera from BNT162b2-vaccinated volunteers against Mu relative to a B.1 isolate [26]. Although the differences observed between these studies may be due to the use of different platforms for the screening of neutralizing antibodies (i.e., pseudovirus or isolates), all these evidenced the resistance of the Mu variant to antibodies elicited by the BNT162b2 vaccine. Nonetheless, further studies evaluating the cell responses to SARS-CoV-2 vaccination must be included to better assess the immunologic effects of SARS-CoV-2 vaccines.

Remarkably, the genomic surveillance and laboratory studies with the emerging lineage B1+L249S+E484K, identified simultaneously with the Mu variant in the same geographic region of Colombia, evidenced reduced neutralization of convalescent sera, accompanied by a decline in cases associated with this variant, demonstrating that this lineage does not represent a concern for public health in Colombia [4]. 

Therefore, the escape of vaccine antibodies and the dramatic increase in cases associated with Mu in Colombia, even with a greater impact than the VOC Gamma, suggest that Mu can be classified as a VOC, depending on the dispersion and global cases in the coming months.

The main limitations of this study are the small sample size (thirty-one patients), and that only serum samples from individuals within 18 and 22 weeks after receiving the second dose of the BNT162b2 vaccine were included. In addition, only the neutralizing antibody response was evaluated.

Finally, this study shows the importance of continuing the surveillance programs of emerging variants, as well as the need to evaluate the resistance of VOCs and VOIs to humoral immunity elicited by the vaccines.

## Figures and Tables

**Table 1:** Genomic characteristics of the viral isolates selected for MN_50_ assays.

Pango Lineage * (WHO Status)	Isolate Name (GISAID ID)	Amino Acid Substitutions
B.1.621 (Mu)	EPI_ISL_1821065	Spike D614G, Spike D950N, Spike E484K, Spike ins145N, Spike N501Y, Spike P681H, Spike R346K, Spike T95I, Spike Y144T, Spike Y145S, N T205I, NSP3 Q57H, NSP3 T237A, NSP3 V256I, NSP3 A562T, NSP3 T720I, NSP4 T492I, NSP6 Q160R, NSP8 P38S, NSP8 Q72R, NSP8 S67F, NSP8 T11K, NSP12 P323L, NSP13 P419S
P.1 (Gamma)	EPI_ISL_2500971	Spike D138Y, Spike D614G, Spike E484K, Spike H655Y, Spike K417T, Spike L18F, Spike N501Y, Spike P26S, Spike R190S, Spike T20N, Spike T1027I, Spike V1176F, N D415G, N G204R, N P80R, N R203K, NSP1 P80L, NSP3 S253P, NSP3 K977Q, NSP3 S370L, NSP3 T1303I, NSP3 V1253F, NSP6 F108del, NSP6 G107del, NSP6 S106del, NSP8 E92K, NSP13 E341D
B.1.111	EPI_ISL_526971	Spike D614G, N M234I, NSP3 Q57H, NSP3 S1285F, NSP12 P323L

* Pango v.3.1.11 2021-08-24. Abbreviations. N: Nucleocapsid, NSP: Non Structural Protein, ins: insertion, del: deletion

**Table 2:** Comparison of MN50 titers and binding antibody units.

Comparison		Spearman r	*p* Value *	95% Confidence Interval
MN50 B.1.111	Anti-S IgG titer BAU/mL	0.7131	<0.0001	0.34715–0.8551
MN50 Gamma (P.1)	Anti-S IgG titer BAU/mL	0.5037	0.004	0.1711–0.7332
MN50 Mu (B.1.621)	Anti-S IgG titer BAU/mL	0.6909	<0.0001	0.4368–0.8429

* Two-tailed.

## Data Availability

All supporting data are included in this manuscript.
